# Changing Patterns in Hospitalisations of Patients with Systemic Lupus Erythematosus over Three Decades at a Tertiary Referral Centre in Catalonia

**DOI:** 10.3390/jcm15093407

**Published:** 2026-04-29

**Authors:** Jesús Cívico-Ortega, Sergio Prieto-González, Olga Araújo, Georgina Espígol-Frigolé, Verónica Gómez-Caverzaschi, Maria Cecilia Garbarino, Ignasi Rodríguez-Pintó, Maria Cinta Cid, Xavier Crespo-Timoner, Rita Reig-Viader, José Hernández-Rodríguez, Gerard Espinosa, Ricard Cervera

**Affiliations:** 1 Department of Autoimmune Diseases, Center of the European Reference Network (ERN) on Rare and Complex Connective Tissue and Musculoskeletal Diseases (ReCONNET) and ERN for Rare Immunodeficiency, Autoinflammatory and Autoimmune Diseases (RITA), Spanish Center of the Centros, Servicios y Unidades de Referencia (CSUR), Catalan Center of the Xarxa d’Unitats d’Expertesa Clínica (XUEC) in Autoinflammatory Diseases and Autoimmune Diseases, Hospital Clínic, 08036 Barcelona, Spain; jesuscivico94@gmail.com (J.C.-O.); sprieto@clinic.cat (S.P.-G.); olaraujo@clinic.cat (O.A.); gespigol@clinic.cat (G.E.-F.); caverzaschi@clinic.cat (V.G.-C.); garbarino@clinic.cat (M.C.G.); irodrip@clinic.cat (I.R.-P.); mccid@clinic.cat (M.C.C.); xcrespo@clinic.cat (X.C.-T.); jhernan@clinic.cat (J.H.-R.); rcervera@clinic.cat (R.C.); 2 Department of Medicine, Faculty of Medicine and Health Sciences, University of Barcelona, 08036 Barcelona, Spain; rviader@clinic.cat; 3 Institut d’Investigacions Biomèdiques August Pi i Sunyer (IDIBAPS), 08036 Barcelona, Spain; 4 Department of Internal Medicine, Hospital Universitario Virgen de la Victoria, 29010 Málaga, Spain; 5 Department of Quality and Clinical Safety, Hospital Clinic de Barcelona, 08036 Barcelona, Spain

**Keywords:** flare, hospitalisation, infection, systemic lupus erythematosus, trends

## Abstract

**Background/Objectives**: Hospitalisations in systemic lupus erythematosus (SLE) reflect disease severity, accumulated damage, and the burden of comorbidity, remaining a major determinant of healthcare utilisation. Recent evidence suggests a shift from flare-driven admissions toward complications related to infections, comorbidities, and long-term treatment effects. We aimed to analyse the causes, characteristics, and outcomes of hospital admissions in patients with systemic lupus erythematosus (SLE) over a 30-year period in a tertiary referral centre in Catalonia (Spain) and to evaluate changes over time and prognostic factors associated with adverse outcomes. **Methods**: A retrospective observational study was conducted including all SLE patients admitted to the Department of Autoimmune Diseases at Hospital Clínic de Barcelona between June 1995 and December 2024. Admissions lasting less than 48 h or lacking clinical documentation were excluded. Variables analysed included demographics, disease duration, comorbidities, cause of admission, treatments, and outcomes. A composite outcome was defined as intensive care unit (ICU) admission, 30-day readmission, or prolonged hospital stay. Statistical analyses included univariate and multivariate regression models. **Results**: Among the 1216 hospital admissions, SLE flares and infections were the most frequent causes. Over the study period, admissions due to infections increased significantly and, in the last five years, exceeded those related to disease flares (33.7% vs. 26.1%). Patients hospitalized for flares were younger and had a shorter disease duration, whereas infection-related admissions were more common among older patients, those with overlap syndromes, and those with higher damage scores. Vascular events and SLE flares were independently associated with poorer outcomes. Although antimalarial use increased over time, it remained suboptimal, largely due to drug toxicity and newly diagnosed cases (from 45.2% to 69.7%; *p* < 0.001). Treatment strategies also evolved, with a shift toward lower glucocorticoid doses (from 14.5% to 38.3%; *p* < 0.001), and mycophenolate mofetil replacing cyclophosphamide as the preferred immunosuppressive agent. **Conclusions**: Hospitalisation patterns in SLE have shifted over time, with infections emerging as the leading cause of admission. This trend reflects an evolving patient profile characterized by older age, greater accumulated damage, comorbidities, and increased exposure to immunosuppressive therapies. These findings underscore the need for optimized infection prevention strategies and individualized treatment approaches to improve outcomes in contemporary SLE care.

## 1. Introduction

Hospitalisations in patients with systemic lupus erythematosus (SLE) reflect inadequate disease control, accumulated organ damage, and a substantial burden of comorbidity [1]. Despite significant advances in diagnosis and therapeutic strategies, SLE continues to impose considerable demands on healthcare systems [2]. Hospital admissions remain frequent and clinically complex, encompassing both disease-related manifestations and complications such as infections, cardiovascular events, and effects of immunosuppressive therapy [3,4,5].

Previous studies from North America [4], Europe [5], and Asia [6,7] have reported annual hospitalisation rates ranging from 9% to 57% among SLE patients. Importantly, a growing proportion of admissions appear to be driven not by active immunological disease alone, but by comorbid conditions, treatment-related complications, and accumulated organ damage. This evolving pattern suggests a shift toward a hospitalised SLE population characterised by lower disease activity but increased clinical vulnerability and long-term complications.

In this context, our group previously analysed 814 hospitalisations in 339 SLE patients admitted to Hospital Clínic de Barcelona between 1995 and 2015, documenting a progressive decline in flare-related admissions alongside an increase in infection-related hospitalisations and damage accrual [8]. These findings were consistent with national-level data reported by Moreno-Torres et al. [9], who identified infections as the second leading cause of hospitalisation among SLE patients in Spain and the cause of 25% of all in-hospital deaths. More recently, Pires et al. [10] further highlighted the growing impact of infections and comorbidities on SLE-related hospitalisations.

We hypothesised that these changes reflect evolving disease management paradigms, including reduced glucocorticoid exposure, the implementation of treat-to-target strategies, wider use of immunosuppressive and biologic therapies, and organisational innovations such as day hospital care for mild-to-moderate flares. As these approaches have become increasingly consolidated over the past decade, it is necessary to reassess whether the trends previously observed at our centre have persisted, stabilised, or further evolved.

Therefore, the present study extends our earlier work by analysing hospitalisations among SLE patients at a tertiary referral centre in Catalonia, Spain over a 30-year period (1995–2024). We aimed to characterise the causes and clinical features of admissions, evaluate changes over time in hospitalisation patterns and outcomes across consecutive 5-year intervals, and identify prognostic factors associated with adverse inpatient outcomes, with the ultimate goal of improving risk stratification and inpatient care in contemporary SLE.

## 2. Materials and Methods

### 2.1. Study Design and Patient Selection

Patients were identified from the hospital’s electronic administrative database, which records discharge diagnoses, by searching for the terms “lupus” or “systemic lupus erythematosus”. Using this approach, we conducted a retrospective observational study including all patients diagnosed with SLE and admitted to the Department of Autoimmune Diseases at Hospital Clínic de Barcelona, a tertiary university hospital, between June 1995 and December 2024. SLE classification was established according to the criteria in use at the time of diagnosis: the 1982 American College of Rheumatology (ACR) criteria [11] and their 1997 revision [12] for patients diagnosed before 2012, the Systemic Lupus International Collaborating Clinics (SLICC) criteria for diagnoses between 2012 and 2019, and the ACR/EULAR 2019 classification criteria [13] thereafter. Associated Sjögren’s disease was identified according to the classification criteria available at the time of diagnosis. The American-European Consensus Group (AECG) criteria [14] were applied for diagnoses made before 2016, including earlier cases reassessed retrospectively according to these criteria, while the ACR/EULAR 2016 classification criteria [15] were used thereafter.

Admissions lasting less than 48 h for intravenous treatment or lacking adequate clinical documentation were excluded. Changes over time were analysed across the entire study period. To this end, hospitalisations were grouped into consecutive 5-year intervals, enabling the evaluation of changes over time in admission causes, clinical characteristics, treatments, and outcomes.

The study was approved by the Institutional Review Board (protocol HCB/2018/1221, approved 17 January 2019). An informed consent was not required given the long-term study period and the retrospective nature of the study.

### 2.2. Data Collection and Variables

Collected variables were obtained through a comprehensive review of patients’ medical records and included demographic characteristics, overlap with other autoimmune diseases, comorbidities (including cardiovascular risk factors), newly diagnosed versus previously established SLE, disease duration (from diagnosis to admission), cause of admission, length of hospital stay, cumulative organ damage assessed using the Systemic Lupus International Collaborating Clinics/American College of Rheumatology Damage Index (SDI) [16], admission to intensive care unit (ICU), hospitalisation outcome, 30-day readmission, and treatments at admission. Glucocorticoid doses were standardised and expressed as prednisone-equivalent doses and categorised as low (≤5 mg/day), moderate (>5 to ≤30 mg/day), or high (>30 mg/day).

For flare-related admissions, additional variables were collected, including disease activity measured by SLE Disease Activity Index 2000 (SLEDAI-2K) [17], flare type (haematological, mucocutaneous, articular, serositis, renal or neuropsychiatric), and relevant laboratory biomarkers (complement levels and anti-double-stranded DNA antibody titres). Causes of admission were considered mutually exclusive; when more than one potential cause was identified, two investigators (J.C.-O. and G.E.) independently reviewed the medical records and reached a consensus.

### 2.3. Statistical Analysis

Normality of continuous variables was assessed using the Kolmogorov–Smirnov test. For normal distributions, data were described as mean (±standard deviation (SD)). Highly skewed data were summarized using median and interquartile range (IQR), with non-parametric tests applied. Categorical variables were reported as absolute and relative frequencies. Differences across decades were analyzed using chi-square for categorical variables and ANOVA or Kruskal–Wallis for continuous variables, as required.

Regression analysis followed a two-step approach. First, univariate models were used as a screening step to identify candidate variables (*p* < 0.2 and/or clinical relevance) for inclusion in the multivariable model. This more permissive threshold is consistent with recommended model-building strategies, which aim to avoid excluding potentially important predictors or confounders at an early stage. Subsequently, forward multivariate modelling was performed. Collinear or unstable variables were excluded. Results were presented as odds ratios (OR) with 95% confidence intervals (CI), and statistical significance was set at *p* < 0.05.

As previously reported by Pires et al. [8], the low frequency of individual adverse events and the presence of collinearity among outcomes limited the statistical power of separate analyses for in-hospital mortality, intensive care unit (ICU) admission, and 30-day readmission. To overcome this limitation, we constructed a composite outcome capturing severe hospitalisation trajectories, defined by the occurrence of any of the following: (i) ICU admission during the index hospitalisation; (ii) unplanned readmission within 30 days of discharge; or (iii) prolonged hospital stay, defined as a length of stay exceeding the 75th percentile (≥10 days) [18,19,20]. This approach, commonly used in observational studies when no standard clinical threshold is available, allows identification of patients with the most resource-intensive admissions. Although these components reflect partially distinct dimensions of severity, their combination increased the number of events and improved the statistical power of the multivariable analyses. All data were processed using IBM SPSS Statistics for Mac, version 29.0.2.0 (IBM Corporation, Armonk, NY, USA).

## 3. Results

**Figure 1:**
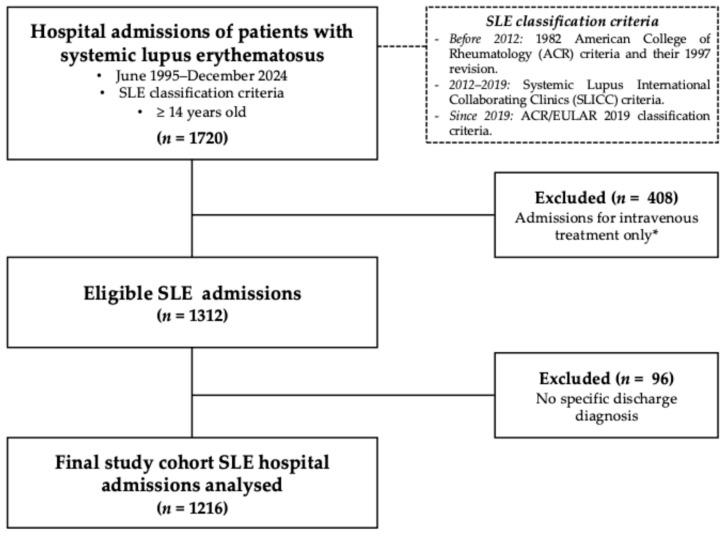
Flowchart of patient selection. * Admissions for scheduled intravenous treatment without an associated acute clinical diagnosis. Abbreviations: ACR: American College of Rheumatology; EULAR: European Alliance of Associations for Rheumatology; IV: intravenous; SLE: systemic lupus erythematosus.

A total of 1720 hospital admissions were initially identified. Of these, 504 (29.3%) were excluded: 408 (23.7%) due to short stays for intravenous therapy and 96 (5.5%) due to insufficient clinical documentation. The final analysis included 1216 admissions involving 500 patients (Figure 1).

### 3.1. General Characteristics

Among the 1216 admissions, 87.4% were women. The mean age at admission was 42.4 years (15.6), and the median disease duration was 8 years (3–17) Overlap with other systemic autoimmune diseases was present in one-third of cases. The median SDI at admission was 0 (0–1). Table 1 summarises the main features of hospitalised SLE patients.

### 3.2. Causes of Hospital Admission

SLE flare was the leading cause of admission (35%), followed by infection (23.4%) and diagnostic procedures (22%) (Table 2). Among flare-related admissions, 12.4% of them were newly diagnosed SLE and renal involvement was predominant (41.1%). Among infection-related cases, respiratory (45.4%), urinary (20.7%), and gastrointestinal (15.4%) sources were the most common.

### 3.3. Subgroup Characteristics and Risk Factors

Subgroup analyses focused on the two most frequent causes of hospital admission: SLE flares and infections (Table 3). Admissions related to diagnostic procedures were excluded because of limited relevance, while thrombotic events were not analysed separately due to their small sample size. Comparative analyses were performed between flare-related and non-flare related admissions, as well as between infection-related and non-infection-related admissions, to better characterise the clinical profiles associated with each cause. Multivariable regression models were subsequently applied to identify factors independently associated with flare- and infection-related hospitalisations (Table 4).

#### 3.3.1. SLE Flare-Related Admissions

Patients admitted for SLE flares were younger (37.4 years (12.9) vs. 45.9 years (16.1)) and had shorter disease duration (median 6 years (1–13) vs. 10 years (4–20)) than those admitted for other causes. This group exhibited lower prevalences of arterial hypertension (19.5% vs. 29.6%) and Sjögren’s disease (15.7% vs. 21%) as well as lower cumulative organ damage as measured by the SDI (although median SDI values were identical, differences in mean ranks were statistically significant on Mann–Whitney testing). Patterns of glucocorticoid use also differed, with low-dose therapy (≤5 mg/day) being less frequent (20.1% vs. 31.1%) and moderate-to-high doses predominating among flare-related admissions. In addition, hospital stays were longer in this subgroup (median 7 days (4–11) vs. 5 days (2–9)).

Univariate analysis identified 30-day readmission as a risk factor for flare-related admission (OR 1.638, 95% CI 1.008–2.661; *p* = 0.046). Protective factors included age (OR 0.965, CI 0.956–0.973; *p* < 0.001), disease duration (OR 0.940, CI 0.926–0.954; *p* < 0.001), SDI (OR 0.640, CI 0.562–0.728; *p* < 0.001), Sjögren’s disease (OR 0.677, CI 0.495–0.925; *p* = 0.014), hypertension (OR 0.570, CI 0.428–0.759; *p* < 0.001), and low-dose glucocorticoids (OR 0.591, CI 0.414–0.844; *p* = 0.004). In multivariate analysis, only age (OR 0.980, CI 0.970–0.989; *p* < 0.001) and disease duration (OR 0.965, CI 0.948–0.981; *p* < 0.001) remained significant.

#### 3.3.2. Infection-Related Admissions

Patients admitted due to infections were older than those admitted for other causes (mean age 48 ± 17.2 vs. 40.7 ± 14.5 years), and had a longer disease duration (median 13 years (6–22.2) vs. 7 years (3–15)), This subgroup showed a higher prevalences of hypertension (34.5% vs. 23.5%), and greater cumulative organ damage, as reflected by higher SDI scores (median 1 (0–2) vs. 0 (0–1)). Overlap syndromes were more frequent (52.1% vs. 38.8%), particularly Sjögren’s disease (29.6% vs. 16%). Use of biologic agent (8% vs. 2.8%) and low-dose glucocorticoids (31.8% vs. 25.9%) were also more common among infection-related admissions; patients receiving biologic therapy for non-SLE indications were excluded from this analysis. ICU admission occurred more frequently in this group (8.3% vs. 4.3%) and hospital stays were slightly longer (median 6 days (4–9) vs. 6 days (2–9)).

In univariate analysis, infection-related admission was associated with older age, longer disease duration, higher SDI scores, overlap syndromes (particularly Sjögren’s disease), arterial hypertension, low-dose glucocorticoid use, and biologic therapy (all *p* ≤ 0.032; odds ratio (OR) range 1.03–2.93). Conversely, azathioprine use and a history of smoking were associated with lower risk of infection-related admission (OR 0.55 and 0.67, respectively). In multivariate analysis, older age, higher SDI, Sjögren’s disease, arterial hypertension, and biologic therapy remained independently associated with infection-related hospitalisation (all *p* ≤ 0.026).

### 3.4. Composite Outcome: ICU Admission, Readmission, and Prolonged Stay

The composite outcome was observed in 377 admissions accounting for 31% of the total hospitalisations. The distribution of individual components was as follows: ICU admission (*n* = 66, 5.4%), 30-day readmission (*n* = 90, 7.4%), and prolonged hospitalisation defined as length of stay exceeding the 75th percentile (≥10 days), which occurred in 293 admissions (24%). Partial overlap among components was observed, with 70 admissions (18.5% of those meeting the composite outcome) fulfilling more than one criterion.

#### 3.4.1. ICU Admission

A total of 66 hospitalisations involving 56 patients required ICU admission. The mean age at admission was 40.3 ± 15.3 years, with a median disease duration of 12.5 (16.2) years. The most frequent causes of ICU admission were infection (37.9%) and SLE flare (36.4%), followed by other autoimmune diseases (9.1%). The median length of hospital stay was 17 days (8–31), and the median SDI at admission was 0.5 (0.5–2). Antimalarial therapy was being used at admission in 51.5% of cases. Among flare-related ICU admissions, the median SLEDAI-2K was 16 (11.7), with renal involvement (75%), haemolytic anaemia (45%), and constitutional symptoms (33.3%) being the most common clinical manifestations.

#### 3.4.2. Readmission

Among the 500 unique patients included in the study, 240 (48%) experienced a single hospitalisation, whereas 260 (52%) had multiple admissions, with a mean of 2.4 ± 2.4 admissions per patient. Ninety patients (18%) were readmitted within 30 days of discharge, most commonly due to SLE flare (36.7%), infection (24.4%), and diagnostic procedures (22.2%).

#### 3.4.3. Mortality

During the study period, 12 in-hospital deaths were recorded, all occurring in women. The leading causes of death were infection (*n* = 5) and catastrophic antiphospholipid syndrome (CAPS, *n* = 4). The mean age at admission was 40.3 ± 11.2 years, with a median disease duration of 14 years (7–22.5). Median length of hospital stay was 18 days (6.25–38.5), and the median SDI at admission was 0 (0–3).

### 3.5. Risk Factors for the Composite Outcome

In univariate analysis, several factors were significantly associated with the composite outcome (30-day readmission, ICU admission, or prolonged hospital stay). These included older age (OR 1.01, 95% CI 1–1.02; *p* = 0.002), higher cumulative organ damage (SDI; OR 1.16, 95% CI 1.05–1.28; *p* = 0.004), the presence of overlap syndrome (OR 2.03, 95% CI 1.58–2.62; *p* < 0.001), APS (OR 1.84, 95% CI 1.4–2.43; *p* < 0.001), arterial hypertension (OR 1.63, 95% CI 1.24–2.15; *p* < 0.001), SLE flare as the cause of admission (OR 1.42, 95% CI 1.1–1.83; *p* = 0.008), and vascular events (OR 3.05, CI 1.80–5.15; *p* < 0.001). Antimalarial therapy was associated with a reduced risk of the composite outcome (OR 0.7, 95% CI 0.54–0.9; *p* = 0.005). In multivariate analysis, overlap syndromes (OR 1.85, CI 1.42–2.40; *p* < 0.001), arterial hypertension (OR 1.55, 95% CI 1.16–2.1; *p* = 0.003), SLE flare (OR 1.77, 95% CI 1.33–2.33; *p* < 0.001), and vascular events (OR 3.11, 95% CI 1.80–5.38; *p* < 0.001) remained independently associated with adverse outcomes, while antimalarial use showed a trend towards protection (OR 0.78, 95% CI 0.60–1.01; *p* = 0.057) (Table 5).

Among admissions due to SLE flares, univariate predictors of the composite outcome included higher disease activity measured by SLEDAI-2K (OR 1.11, 95% CI 1.07–1.15; *p* < 0.001), newly diagnosed SLE (OR 1.89, 95% CI 1.05–3.4; *p* = 0.033), constitutional symptoms (OR 1.53, 95% CI 1.01–2.30; *p* = 0.043), haemolytic anaemia (OR 2.20, CI 1.28–3.79; *p* = 0.004), renal flare (OR 2.60, 95% CI 1.71–3.94; *p* < 0.001), and neuropsychiatric flare (OR 4.37, 95% CI 1.62–11.75; *p* = 0.004). In the multivariable model, SLEDAI-2K (OR 1.06, 95% CI 1.00–1.11; *p* = 0.036), APS (OR 1.84, 95% CI 1.13–2.3; *p* = 0.015), constitutional symptoms (OR 1.65, 95% CI 1.02–2.67; *p* = 0.043), haemolytic anaemia (OR 2.15, 95% CI 1.16–3.93; *p* = 0.014), renal flare (OR 2.45, 95% CI 1.41–4.23; *p* = 0.001), and neuropsychiatric flare (OR 5.70, 95% CI 1.8–18.55; *p* = 0.003) remained independently associated with the composite outcome (Table 5).

In contrast, among infection-related admissions, arterial hypertension was the only variable consistently associated with the composite outcome in both univariate (OR 2.14, 95% CI 1.26–3.63; *p* = 0.004) and multivariate analysis (OR 2.26, 95% CI 1.29–3.96; *p* = 0.004).

### 3.6. Changes over 30 Years

**Figure 2:**
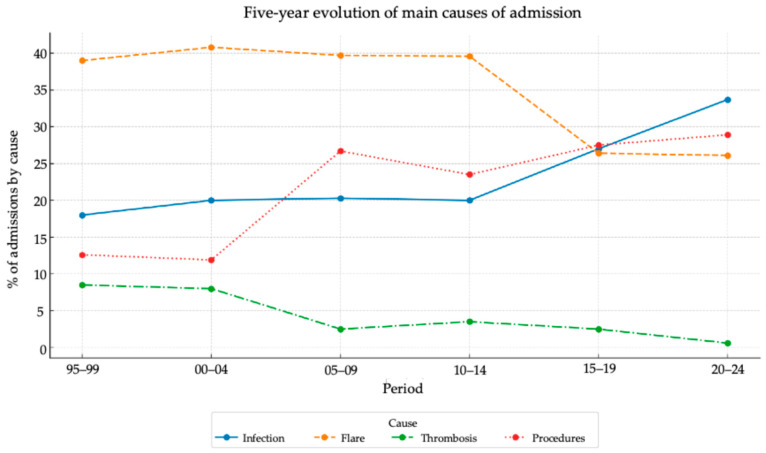
Five-year trends in the main causes of hospital admission.

The overall number of hospital admissions remained stable throughout the study period (Supplementary Figure S1) with approximately 400 admissions per decade. However, the causes of admission changed substantially over time (Figure 2 and Supplementary Table S1). Infection-related admissions increased significantly, from 19.9% to 31.3% (*p* < 0.001), as did admissions for diagnostic procedures (from 11.9% to 26.7%; *p* < 0.001). In contrast, admissions due to SLE flares declined markedly (from 40.0% to 25.2%; *p* < 0.001) as did those related to thrombotic events (from 8.2% to 1.4%; *p* < 0.001). Notably, during the final five-year period, infections surpassed disease flares as the leading cause of hospitalisation (33.7% vs. 26.1%) (Figure 2).

Patients admitted in the most recent decade (2015–2024) were older (mean age 44.9 vs. 40.8 years; *p* < 0.001) and had a longer disease duration (median 10 vs. 7 years; *p* < 0.001). The prevalence of arterial hypertension decreased over time (from 31.3% to 22.6%; *p* = 0.012), whereas a history of smoking became more common (from 16.1% to 23.3%; *p* = 0.003). Disease activity at admission, measured by SLEDAI-2K, increased modestly (median 8 to 10; *p* = 0.005), while overlap with APS declined (from 24% to 12.7%; *p* = 0.01) (Table 6).

Treatment patterns evolved considerably over the study period. Use of antimalarials increased from 45.2% to 69.7%, immunosuppressive therapies from 27.8% to 51.9%, and biologic agents from 0% to 10.2% (all *p* < 0.001). Mycophenolate mofetil use rose substantially (from 4.9% to 27.4%; *p* < 0.001), replacing cyclophosphamide (from 8.7% to 2.2%). Glucocorticoid prescribing shifted towards lower doses, with low-dose use increasing from 14.5% to 38.3% (*p* < 0.001) and moderate-to-high doses decreasing from 61% to 34.7% (*p* < 0.001). Median length of hospital stays shortened over time (from 6 to 5 days; *p* < 0.001), although ICU admissions became more frequent (from 3.5% to 7.7%; *p* = 0.020) (Table 6).

## 4. Discussion

This comprehensive, 30-year analysis reveals a fundamental shift in SLE hospitalisation epidemiology. Infections have surpassed flares as the leading cause of admission, and this trend is associated with two distinct patient phenotypes. Younger patients with a recent SLE diagnosis or active disease present with high-intensity flares and worse in-hospital outcomes, while older multimorbid patients experience infections and accumulate permanent organ damage. We identified vascular events and renal involvement as the strongest predictors of adverse in-hospital outcomes, with implications for targeted prevention strategies.

Several therapeutic and organisational changes coincided with these epidemiological shifts during the study period. However, causality cannot be established in this observational design. These include: (i) the earlier and more aggressive use of immunosuppressive agents (particularly mycophenolate); (ii) broader access to biologic therapies; (iii) a gradual reduction in glucocorticoid doses aligned with treat-to-target strategies; and (iv) the implementation of day hospital care for mild-to-moderate flare-ups at our institution. While these interventions may have reduced hospitalisations related to flares, they may have inadvertently increased the susceptibility of highly immunosuppressed patients to infections, resulting in the observed shift towards infection-predominant admissions.

Compared with our previous analysis of 814 admissions between 1995 and 2015 [10], this expanded cohort almost doubles the sample size to 1216 admissions and extends the follow-up period by nine years to include 2024, incorporating the era of widespread biologic use and formalised treat-to-target strategies. This additional decade provides novel insights into the long-term impact of therapeutic intensification on hospitalisation patterns.

Few recent studies have examined hospitalisation trends in SLE using large cohorts. Pires et al. [10] observed a non-significant increase in infection-related admissions over time, highlighting the need for updated, large-scale analyses. Previous national registry-based studies [21,22] have described a gradual decline in admissions due to active SLE, with a parallel increase in hospitalisations related to infections and cardiovascular comorbidity. However, most of these analyses relied on administrative data and lacked detailed clinical characterization. Our results confirm these observations and incorporate validated measures of disease activity and accumulated damage.

These evolving patterns call for a redefinition of inpatient care for SLE patients. Future clinical practice must emphasize comorbidity management, infectious disease expertise, and integration of geriatric principles into autoimmune care.

Notably, the relatively low prevalence of certain chronic comorbidities in our cohort may appear unexpected. This finding may be partly explained by the relatively young age of the population, as well as by the characteristics of a specialised autoimmune diseases unit, where hospitalisations are more frequently driven by disease activity and immune-related complications. In addition, patients with significant chronic comorbidities may have been admitted to other specialised departments, potentially leading to an underrepresentation of these conditions in our cohort.

Although a decline in hospitalisations might be expected due to improved flare management and expanded outpatient care, this has been offset by rising admissions for infections and diagnostic procedures, as well as an increasing number of complex referrals to our unit.

Antimalarials increased significantly over time (45.2% to 53.4% to 69.7%; *p* < 0.001), reflecting growing awareness of their protective role in SLE. This trend is consistent with current recommendations [23] and may have contributed to the observed changes in disease expression and hospitalisation patterns, though current rates remain suboptimal. Non-prescription at admission was primarily due to newly diagnosed SLE (11% of flare-related admissions) and drug toxicity, mainly ocular. Excluding new-onset cases, usage reached almost 80% (75–79% per patient), aligning with other cohorts (80–85%) [5,7]. As a national/European referral centre managing complex patients with contraindications, our cohort likely experiences higher cumulative toxicity rates (particularly retinopathy) [24], explaining lower prescription rates compared to less specialized centres and contributing to increased hospitalisation risk.

Glucocorticoid use has evolved notably. Although overall usage rates have changed minimally, dosing patterns reflect a shift toward safer strategies. The rise in low-dose prescriptions (>0 to ≤5 mg/day) and decline in higher doses (>5 mg/day) indicate increased awareness of long-term toxicity and a preference for steroid-sparing approaches. This trend is supported by earlier use of immunosuppressants and biologics, enabling better disease control with reduced steroid exposure [25]. These changes align with international SLE management guidelines [23] and the treat-to-target strategy [26], promoting a balance between disease control and treatment-related harm [27]. Notably, low-dose glucocorticoids were less common in flare-related admissions (consistent with their protective role), while higher doses were more frequent, likely reflecting outpatient management failure in moderate-to-severe flares. Moreover, low-dose glucocorticoid use (31.8% vs. 25.9%) was more frequent among infection-related admissions. This finding may be explained by the age differences between patients receiving >0 to ≤5 mg/day of glucocorticoids (mean age 46.3 years) and those receiving >5 mg/day (39.5 years). Therefore, the group treated with >0 to ≤5 mg/day likely includes patients with long-standing glucocorticoid exposure, who tend to be older and present a higher burden of comorbidities.

Immunosuppressive therapy has also shifted, with mycophenolate emerging as the most commonly used agent and cyclophosphamide declining. This reflects institutional policy favouring mycophenolate for its safety and efficacy, particularly in non-life-threatening scenarios and in women with reproductive plans [28]. Cyclophosphamide has been largely replaced by mycophenolate in membranoproliferative lupus nephritis without poor prognostic markers, and by rituximab in severe or refractory cases, especially in women of childbearing age [23,29].

Interestingly, azathioprine use and smoking history were associated with a lower risk of infection-related admissions in univariate analysis; however, these findings should be interpreted with caution. They likely reflect unmeasured confounding or indication bias inherent to retrospective observational studies. Azathioprine is commonly used as a maintenance therapy in patients with more stable disease, whereas more potent immunosuppressive agents or biologics—typically associated with higher infection risk—are preferentially prescribed in patients with more severe or refractory disease. Similarly, the apparent protective association with smoking is unlikely to represent a true biological effect and may instead be explained by differences in patient profiles or other unmeasured variables. Importantly, neither variable remained significant in multivariate analysis, suggesting that they do not represent independent predictors of infection-related admissions.

Among risk factors for poor outcomes, vascular events were most strongly associated with the composite outcome, underscoring the need for aggressive outpatient prevention strategies [30]. Vascular events in this context should be interpreted as multifactorial. Although APS is a major contributor, these events may also reflect the impact of traditional cardiovascular risk factors such as hypertension, dyslipidaemia, and diabetes mellitus, or a combination of both mechanisms. Given the retrospective design and the frequent overlap between APS-related thrombosis and atherosclerotic disease, it was not always possible to attribute each event to a single underlying cause. SLE flare, particularly renal involvement, was the second most significant predictor, highlighting the importance of early, intensive immunosuppressive therapy. Although antimalarial use did not reach statistical significance, its protective trend reinforces the need for universal prescription in eligible patients.

Although there was an increase in ICU admissions from 3.5% to 7.7% (*p* = 0.020) throughout the study period, in-hospital mortality remained stable. This apparent paradox may reflect several non-mutually exclusive factors: (1) infections are the leading cause of ICU admission and often require intermediate-level care (e.g., monitoring and oxygen therapy), but have a lower mortality rate than catastrophic events; (2) over time, there has been a relaxation of ICU admission criteria, particularly for elderly patients or those not considered to be intubation candidates, which has expanded access to supportive care [31]; (3) referrals from other institutions have increasingly involved critically ill patients who already require ICU-level management upon arrival; and (4) improved critical care practices and antimicrobial stewardship has reduced ICU mortality. Conversely, excluding SARS-CoV-2 patients admitted to other departments may have reduced bias related to the pandemic.

The association between infection-related admissions and overlap syndromes, particularly Sjögren’s disease, is noteworthy. Although not previously reported in this context, Sjögren’s disease is known to increase infection risk due to impaired mucosal barrier function, contributing to excess mortality in isolated cases [32]. Beyond infection risk, the interaction between Sjögren’s disease and SLE may also influence the overall disease course. Patients with overlapping Sjögren’s disease may exhibit distinct clinical and immunological features, including differences in disease activity and autoantibody profiles, particularly anti-Ro/SSA and anti-La/SSB antibodies. These factors may modulate disease expression and outcomes in SLE [33]. However, detailed serological data were not systematically available in our dataset, and we were therefore unable to explore the impact of specific autoantibody profiles on disease activity or hospitalisation outcomes. This represents a potential area for further research.

As with any retrospective study, limitations exist. Patient selection was restricted to admissions within the Department of Autoimmune Diseases, potentially excluding cases managed in other departments (e.g., acute coronary syndrome, stroke, SARS-CoV-2, fractures). Moreover, data were derived from a single centre, which may limit the generalisability of the findings to other institutions, healthcare systems, or geographical settings. Differences in clinical practice, hospital admission policies, and healthcare organisation may influence the observed patterns. Additionally, as a tertiary referral centre, our institution may include a higher proportion of complex cases, potentially introducing selection bias. COVID-19 pandemic may also represent a limitation of our study as it likely introduced bias at different levels, including admissions to different units, delay in seeking medical care due to fear of infection and modifications of healthcare delivery. Furthermore, preventive strategies against infections, such as vaccination programmes, Pneumocystis jirovecii prophylaxis, and screening or treatment for latent tuberculosis, were not systematically recorded and could not be analysed. Changes in these strategies over time may have influenced the observed trends in infection-related hospitalisations and therefore represent a potential source of unmeasured confounding. Additionally, causes of admission were operationalised as mutually exclusive categories. In cases with more than one plausible reason for hospitalisation, records were reviewed by two investigators (J.C.O. and G.E.) and classified by consensus. Although this approach improved consistency, it may oversimplify the multifactorial nature of some admissions, and a degree of misclassification cannot be excluded.

Several factors may have contributed to bias and residual confounding in this 30-year, single-centre SLE cohort analysis. Admissions to other departments (e.g., cardiology for acute coronary syndrome or neurology for stroke) were excluded, which enriched the cohort for autoimmune-focused hospitalisations. Here, the apparent rise in infection-related admissions may reflect increased hospitalisation rates rather than true outpatient infection incidence. Temporal diagnostic shifts (e.g., interpreting elevated CRP as infection rather than a mild flare in recent years), improved electronic health record documentation detecting more comorbidities and confounding by indication (intensive immunosuppression being prescribed for more severe disease) may also independently explain the observed trends, apart from true epidemiological changes.

Changes in classification criteria over time, as well as evolving hospital admission policies and improvements in outpatient management, may have influenced both the case mix and the observed changes over time. In particular, the increasing use of day hospital care and more restrictive admission practices in recent years may have contributed to a shift in the profile of hospitalised patients towards more complex and severe cases.

Nonetheless, the strengths of this study include a large cohort of 1216 admissions from 500 patients, a 30-year longitudinal follow-up by the same department (often the same physicians), which ensures diagnostic and therapeutic homogeneity and minimises interobserver variability. Standardised indices (ACR/EULAR, SLEDAI-2K and SDI), clear exposure and outcome definitions, decade-by-decade analyses and data from a national reference centre enhance the study’s internal validity and provide a robust real-word insight into trends in SLE hospitalisation.

## 5. Conclusions

Over the past three decades, patterns of hospitalisation for SLE have shifted markedly. Infections have now surpassed flares as the leading cause, reflecting two distinct patient profiles: younger patients with high activity flares and older patients with multiple comorbidities who are on chronic immunosuppression. Admissions for SLE flares, overlap syndromes, vascular events, and arterial hypertension are associated with poorer outcomes. These findings highlight the need to redefine inpatient care, with focus on infection prevention, management of comorbidities, and early intervention for high-risk phenotypes.

## Figures and Tables

**Table 1:** Baseline characteristics of patients with SLE and hospital admissions.

Variable	Value
**Study population**	
Female sex ^†^, *n* (%)	437 (87.4)
Age at SLE diagnosis ^†^, years mean (SD)	31.2 (14.8)
Age at hospital admission ^‡^, years mean (SD)	42.4 (15.6)
Disease duration at admission ^‡^, years median (IQR)	8 (3–17)
Newly diagnosed SLE ^‡^, *n* (%)	56 (4.6)
**Cardiovascular risk factors ^†^,** *n* (%)	
Arterial hypertension	317 (26.1)
Smoking history	264 (21.7)
Dyslipidaemia	166 (13.7)
Diabetes mellitus	50 (4.1)
**Associated autoimmune diseases ^†^,** *n* (%)	
Any overlap syndrome	167 (33.4)
Antiphospholipid syndrome	88 (17.6)
Sjögren’s disease	67 (13.4)
**Disease status at admission**	
SDI ^‡^, median (IQR)	0 (0–1)
SLEDAI-2K at admission ^§^, median (IQR)	8 (5–13)
**Biomarkers at admission** ^§^, ***n*** (%)	
Elevated anti-dsDNA	284 (85.8)
Complement consumption	271 (77.2)
Proteinuria > 0.5 mg/24 h	221 (56.5)
**Source of admission ^‡^**, *n* (%)	
Emergency department	517 (42.5)
Elective admission from home	334 (27.5)
Day hospital	152 (12.5)
Outpatient clinic	145 (11.9)
Other hospital department	51 (4.2)
Intensive care unit	17 (1.4)
**Treatment at admission ^‡^, *n* (%)**	
Antimalarials	697 (57.3)
Immunosuppressants	516 (42.4)
Biologic agents	50 (4.2)
Glucocorticoids	950 (78.1)
Anticoagulants	178 (14.6)
Antiplatelet agents	248 (20.4)
**Outcomes ^‡^**	
Length of hospital stay, days median (IQR)	6 (2–9)
30-day readmission, *n* (%)	90 (7.4)
ICU admission, *n* (%)	66 (5.4)
In-hospital mortality, *n* (%)	12 (1.0)

^†^ Calculated per patient (*n* = 500). ^‡^ Calculated per admission (*n* = 1216). ^§^ Calculated per admission in patients admitted due to SLE flare. Abbreviations: dsDNA, double-stranded DNA; IQR, interquartile range; SD, standard deviation; SLE, systemic lupus erythematosus; SLEDAI, SLE Disease Activity Index; SDI, Systemic Lupus International Collaborating Clinics Damage Index.

**Table 2:** Causes of hospital admission in patients with SLE.

Cause of Admission	*n* (%)
**SLE flare**	**426 (35.0)**
Renal involvement	175 (41.1) ^†^
Constitutional symptoms	167 (39.2) ^†^
Haematological	149 (35.0) ^†^
Arthritis	143 (33.6) ^†^
Mucocutaneous involvement	113 (26.5) ^†^
Serositis	79 (18.5) ^†^
Neuropsychiatric involvement	19 (4.5) ^†^
**Infection**	**284 (23.4)**
Respiratory	129 (45.4) ^†^
Urinary	59 (20.7) ^†^
Gastrointestinal	44 (15.4) ^†^
Skin and soft tissue	20 (7.0) ^†^
Other	32 (11.2) ^†^
**Diagnostic procedures**	**267 (22.0)**
Kidney biopsy	232 (86.8) ^†^
**Thrombotic events**	**50 (4.6)**
Arterial	28 (56.0) ^†^
Stroke	12 (42.8) ^‡^
Acute coronary syndrome	6 (21.4) ^‡^
Digital ischemia	6 (21.4) ^‡^
Venous	22 (44.0) ^†^
Deep venous thrombosis	14 (63.6) ^‡^
Pulmonary embolism	5 (22.7) ^‡^
**Musculoskeletal disorders**	**35 (2.9)**
Vertebral fracture	10 (28.5) ^†^
Avascular femoral head necrosis	5 (14.2) ^†^
Glucocorticoid-induced myopathy	3 (8.5) ^†^
**Other causes**	**154 (12.6)**
Other systemic autoimmune disease	34 (2.8) ^†^
Neurological disorders	24 (2.0) ^†^
Cardiovascular disease (non-thrombotic)	24 (2.0) ^†^
Others	72 (5.9) ^†^

^†^ Percentages calculated using the total number of admissions within each main category as de-nominator. ^‡^ Percentages calculated using the total number of admissions within the corresponding thrombotic subgroup as denominator. Abbreviations: SLE: systemic lupus erythematosus.

**Table 3:** Characteristics of subgroups: SLE flare and infection.

Variable	SLE Flare (*n* = 426)	Non-SLE Flare (*n* = 790)	*p*	Infection (*n* = 284)	Non-Infection (*n* = 932)	*p*
Gender, female, *n* (%)	379 (89)	713 (90.3)	0.480	262 (92.3)	830 (89.1)	0.119
Age at SLE diagnosis, years, mean (SD)	29.3 (13.3)	32.2 (15.4)	<0.001 *	33.1 (16.4)	30.6 (14.1)	0.023 *
Age at hospitalisation, years, mean (SD)	37.4 (12.9)	45.9 (16.1)	<0.001 *	48 (17.2)	40.7 (14.5)	<0.001 *
Duration of SLE, years, median (IQR)	6 (1–13)	10 (4–20)	<0.001 *	13 (6–22.2)	7 (3–15)	<0.001 *
Cardiovascular risk factors, *n* (%)						
Arterial hypertension	83 (19.5)	234 (29.6)	<0.001 *	98 (34.5)	219 (23.5)	<0.001 *
Smoking history	87 (20.4)	177 (22.4)	0.424	48 (16.9)	216 (23.3)	0.025 *
Dyslipidaemia	51 (12)	115 (14.6)	0.345	40 (14.1)	126 (13.5)	0.835
Diabetes mellitus	13 (3.1)	37 (4.7)	0.172	17 (6)	33 (3.5)	0.069
Associated autoimmune diseases, *n* (%)						
Overlap	168 (39.4)	342 (43.3)	0.194	148 (52.1)	362 (38.8)	<0.001 *
Antiphospholipid syndrome	118 (27.7)	180 (22.8)	0.057	70 (24.6)	228 (24.5)	0.950
Sjögren’s disease	67 (15.7)	166 (21)	0.025 *	84 (29.6)	149 (16)	<0.001 *
SDI at admission, median (IQR)	0 (0–1)	0 (0–1)	<0.001 *^a^	1 (0–2)	0 (0–1)	<0.001 *
Treatment, *n* (%)						
Antimalarials	246 (57.7)	451 (57.1)	0.825	157 (55.3)	540 (57.9)	0.428
Immunosuppressants	168 (39.4)	348 (44.1)	0.120	119 (41.9)	397 (42.6)	0.836
Biologic agents ^b^	12 (2.8)	36 (4.6)	0.126	22 (8)	26 (2.8)	<0.001 *
Glucocorticoids	327 (76.8)	623 (78.9)	0.398	231 (81.3)	719 (77.1)	0.135
> 0 ≤ 5 mg/24 h	82 (20.1)	234 (31.1)	<0.001 *	88 (31.8)	228 (25.9)	<0.001 *
>5 mg/24 h	226 (55.5)	351 (46.7)	<0.001 *	136 (49.1)	441 (50)	<0.001 *
Anticoagulants	45 (10.6)	133 (16.8)	0.003 *	53 (18.7)	125 (13.4)	0.028 *
Antiplatelets	76 (17.8)	172 (21.8)	0.201	68 (23.9)	180 (19.3)	0.207
Outcomes ^c^						
Days of admission, median (IQR)	7 (4–11)	5 (2–9)	<0.001 *	6 (4–9)	6 (2–9)	0.030 *
Readmission at 30 days, *n* (%)	29 (7)	41 (5.3)	0.271	20 (7.3)	50 (5.5)	0.271
Need for admission in ICU, *n* (%)	24 (5.6)	39 (5)	0.685	23 (8.3)	40 (4.3)	0.009 *
Mortality ^d^, *n* (%)	3 (0.7)	8 (1)	0.756	5 (1.8)	6 (0.6)	0.140

^a^ Statistically significant differences were found in the Mann–Whitney test despite identical medians and interquartile ranges, as the test evaluates differences in mean ranks. Patients admitted for SLE flare tended to have lower SDI scores. ^b^ Patients receiving biologic therapy for other diseases, such as psoriasis or Still’s disease, were excluded from the ‘Biologics agents’ group. ^c^ Patients receiving biologic therapy for other diseases, such as psoriasis or Still’s disease, were excluded from the ‘Outcomes’ group. Patients readmitted at 30 days for diagnostic procedures were also excluded from ‘Readmission at 30 days group’. ^d^ Statistical values calculated by Fisher’s test. * *p*-values < 0.05 were considered statistically significant. Abbreviations: ICU: intensive care unit; IQR: interquartile range; SD: standard deviation; SLE: systemic lupus erythematosus; SDI: Systemic Lupus International Collaborating Clinics Damage Index.

**Table 4:** Risk factors for SLE flare admission and infection admission events by regression.

	Univariate Analysis		Multivariate Analysis ^a^	
Variable	OR (CI 95%)	*p*	OR (CI 95%)	*p*
**SLE Flare as cause of admission**				
Gender (female)	1.173 (0.793–1.735)	0.425	1.111 (0.733–1.684)	0.619
Age at SLE diagnosis	0.985 (0.977–0.993)	<0.001 *	NS	NS
Age at admission	0.965 (0.956–0.973)	<0.001 *	0.980 (0.970–0.989)	<0.001 *
SDI	0.640 (0.562–0.728)	<0.001 *	0.815 (0.708–0.939)	0.005 *
Duration of SLE, years	0.940 (0.926–0.954)	<0.001 *	0.965 (0.948–0.981)	<0.001 *
Sjögren’s disease	0.677 (0.495–0.925)	0.014 *	1.030 (0.725–1.464)	0.868
Present admission is a 30-day readmission	1.638 (1.008–2.661)	0.046 *	1.491 (0.879–2.530)	0.138
Arterial hypertension	0.570 (0.428–0.759)	<0.001 *	0.845 (0.610–1.170)	0.310
Anticoagulants	0.566 (0.394–0.813)	0.002 *	0.830 (0.556–1.238)	0.360
Glucocorticoids > 0 ≤ 5 mg/24 h	0.591 (0.414–0.844)	0.004 *	0.727 (0.497–1.063)	0.100
**Infection as cause of admission**				
Gender (female)	0.652 (0.395–1.076)	0.094	0.749 (0.445–1.261)	0.276
Age at SLE diagnosis	1.012 (1.003–1.021)	0.008 *	NS	NS
Age at admission	1.030 (1.021–1.039)	<0.001 *	1.015 (1.005–1.025)	0.004 *
SDI	1.588 (1.428–1.767)	<0.001 *	1.401 (1.248–1.573)	<0.001 *
Duration of SLE, years	1.051 (1.037–1.066)	<0.001 *	NS	NS
Overlap	1.718 (1.310–2.253)	<0.001 *	NS	NS
Sjögren’s disease	2.259 (1.655–3.084)	<0.001 *	1.513 (1.075–2.131)	0.018 *
Arterial hypertension	1.799 (1.344–2.408)	<0.001 *	1.442 (1.046–1.990)	0.026 *
Smoking history	0.672 (0.474–0.953)	0.026 *	NS	NS
Obesity	2.051 (1.041–4.043)	0.038 *	NS	NS
Azathioprine	0.553 (0.347–0.881)	0.013 *	0.690 (0.423–1.123)	0.135
Biologic agents	2.932 (1.634–5.260)	<0.001 *	1.825 (1.269–2.624)	0.001 *
Anticoagulants	1.513 (1.061–2.157)	0.022 *	NS	NS
Glucocorticoids > 0 ≤ 5 mg/24 h	1.541 (1.038–2.287)	0.032 *	NS	NS

^a^ Colinear variables or those which introduced instability to the model were excluded from the multivariable analysis. * *p*-values < 0.05 were considered statistically significant. Patients receiving biologic therapy for other diseases, such as psoriasis or Still’s disease, were excluded from the analysis. Abbreviations: CI: confidence interval; NS: non-statistical significance; OR: odds ratio; SLE: systemic lupus erythematosus; SDI: Systemic Lupus International Collaborating Clinics Damage Index.

**Table 5:** Risk factors for composite outcome (30-day readmission or ICU admission or prolonged hospital stay (above the 75th percentile) in the whole sample, in SLE flare admissions and in infection admissions calculated by univariate and multivariate regression.

Sample	Univariate Analysis		Multivariate Analysis ^a^	
	OR (CI 95%)	*p*	OR (CI 95%)	*p*
**Composite outcome in the whole sample**				
Gender (female)	1.242 (0.829–1.861)	0.292	1.231 (0.811–1.867)	0.335
Age at SLE diagnosis	1.013 (1.005–1.022)	0.002 *	NS	NS
Age at admission	1.010 (1.002–1.018)	0.011 *	1.000 (1.000–1.016)	0.324
SDI	1.159 (1.049–1.281)	0.004 *	NS	NS
Overlap	2.034 (1.581–2.618)	<0.001 *	1.852 (1.422–2.405)	<0.001 *
Antiphospholipid syndrome	1.842 (1.396–2.431)	<0.001 *	NS	NS
Diabetes mellitus	1.966 (1.104–3.502)	0.022 *	NS	NS
Arterial hypertension	1.635 (1.243–2.151)	<0.001 *	1.553 (1.164–2.099)	0.003 *
Dyslipidaemia	1.436 (1.016–2.030)	0.040 *	NS	NS
SLE Flare as cause of admission	1.417 (1.096–1.833)	0.008 *	1.768 (1.334–2.331)	<0.001 *
Vascular events ^b^	3.050 (1.804–5.155)	<0.001 *	3.112 (1.802–5.382)	<0.001 *
Antimalarials	0.699 (0.545–0.898)	0.005 *	0.784 (0.605–1.011)	0.057
Anticoagulants	1.649 (1.182–2.300)	0.003 *	NS	NS
**Composite outcome in SLE flare admissions**				
Gender (female)	0.746 (0.457–1.753)	0.671	0.683 (0.322–1.453)	0.321
SLEDAI-2K at admission	1.109 (1.068–1.151)	<0.001 *	1.056 (1.005–1.114)	0.036 *
Newly diagnosed SLE	1.891 (1.052–3.399)	0.033 *	0.926 (0.454–1.877)	0.813
Antiphospholipid syndrome	1.594 (1.024–2.482)	0.039 *	1.837 (1.126–2.998)	0.015 *
Constitutional symptoms	1.527 (1.013–2.303)	0.043 *	1.653 (1.024–2.673)	0.043 *
Haemolytic anaemia	2.203 (1.280–3.790)	0.004 *	2.147 (1.164–3.931)	0.014 *
Renal flare	2.600 (1.715–3.943)	<0.001 *	2.448 (1.413–4.230)	0.001 *
Neuropsychiatric flare	4.366 (1.623–11.748)	0.004 *	5.700 (1.799–18.554)	0.003 *
Proteinuria	1.730 (1.120–2.614)	0.014 *	NS	NS
Antimalarials	0.654 (0.435–0.984)	0.042 *	0.698 (0.430–1.101)	0.119
**Composite outcome in infection admissions**				
Gender (female)	0.712 (0.250–2.026)	0.524	0.620 (0.265–2.208)	0.765
Age at admission	1.003 (0.988–1.017)	0.732	0.995 (0.979–1.011)	0.525
Arterial hypertension	2.148 (1.269–3.634)	0.004 *	2.263 (1.293–3.962)	0.004 *

^a^ Colinear variables or those which introduced instability to the model were excluded from the multivariable analysis. ^b^ Defined as thrombotic events of all types, whether they are cause of admission or not. * *p*-values < 0.05 were considered statistically significant. Patients receiving biologic therapy for other diseases, such as psoriasis or Still’s disease, were excluded from the analysis. Abbreviations: CI: confidence interval; NS: non-statistical significance; OR: odds ratio; SLE: systemic lupus erythematosus; SLEDAI: SLE Disease Activity Index; SDI: Systemic lupus International Collaborating Clinics Damage Index.

**Table 6:** Decadal trends in hospitalized patients with systemic lupus erythematosus.

Variable	1995–2004	2005–2014	2015–2024	*p*
Included admissions (number of patients)	403 (196)	401 (139)	412 (165)	0.892
Excluded admissions due to missing data, *n* (%)	36 (8.9)	30 (7.5)	30 (7.3)	0.641
Gender, female, *n* (%)	173 (88.3)	121 (87.1)	143 (86.7)	0.890
Age at SLE diagnosis, years, mean (SD)	32.3 (14.5)	29.4 (13.5)	31.9 (15.9)	0.014 *
Age at hospitalisation, years, mean (SD)	41.3 (15.3)	40.8 (14.3)	44.9 (16.6)	<0.001 *
Duration of SLE, years, median (IQR)	7 (2–14)	8 (6–12)	10 (4–21)	<0.001 *
Cardiovascular risk factors, *n* (%)				
Arterial hypertension	126 (31.3)	98 (24.4)	93 (22.6)	0.012 *
Smoking history	65 (16.1)	103 (25.7)	96 (23.3)	0.003 *
Dyslipidaemia	64 (15.9)	57 (14.2)	45 (10.9)	0.178
Diabetes mellitus	18 (4.5)	10 (2.5)	22 (5.3)	0.113
Associated autoimmune diseases, *n* (%)				
Overlap	72 (36.7)	36 (25.9)	59 (35.8)	0.860
Antiphospholipid syndrome	47 (24)	20 (14.4)	21 (12.7)	0.01 *
SDI at admission, median (IQR)	0 (0–1)	0 (0–1)	0 (0–2)	0.008 *
SLEDAI-2K at admission ^a^, median (IQR)	8 (5–12)	8 (6–12)	10 (6–17.5)	0.005 *
Treatment, *n* (%)				
Antimalarials	182 (45.2)	214 (53.4)	287 (69.7)	<0.001 *
Immunosuppressants	112 (27.8)	190 (47.4)	214 (51.9)	<0.001 *
Mycophenolate	17 (4.2)	96 (23.9)	113 (27.4)	<0.001 *
Methotrexate	12 (3)	22 (5.5)	30 (7.3)	0.022 *
Cyclophosphamide	35 (8.7)	20 (5)	9 (2.2)	<0.001 *
Biologic agents ^b^	0	7 (1.7)	41 (10.2)	<0.001 *
Glucocorticoids	318 (78.9)	331 (82.5)	301 (73.1)	0.004 *
>0 and ≤5 mg/24 h	50 (14.5)	108 (26.9)	158 (38.3)	<0.001 *
>5 mg/24 h	211 (61)	223 (55.6)	143 (34.7)	<0.001 *
Anticoagulants	50 (12.4)	55 (13.7)	73 (17.7)	0.082
Antiplatelets	75 (18.6)	73 (18.2)	100 (24.3)	0.098
Outcomes ^c^				
Days of admission, median (IQR)	6 (4–10)	5 (2–9)	5 (2–9)	<0.001 *
Readmission at 30 days, *n* (%)	25 (6.3)	26 (6.7)	19 (4.8)	0.498
Need for admission in ICU, *n* (%)	14 (3.5)	18 (4.5)	31 (7.7)	0.020 *
Mortality, *n* (%)	4 (1)	4 (1)	3 (0.7)	0.909

^a^ Patients admitted due to SLE flare. ^b^ Patients receiving biologic therapy for other diseases, such as psoriasis or Still’s disease, were excluded from the ‘Biologics agents’ group. ^c^ Patients receiving biologic therapy for other diseases, such as psoriasis or Still’s disease, were excluded from the ‘Outcomes’ group. Patients readmitted at 30 days for diagnostic procedures were also excluded from ‘Readmission at 30 days group’. * *p*-values < 0.05 were considered statistically significant. Abbreviations: ICU: intensive care unit; IQR: interquartile range; SD: standard deviation; SLE: systemic lupus erythematosus; SLEDAI: SLE Disease Activity Index; SDI: Systemic Lupus International Collaborating Clinics Damage Index.

## Data Availability

Data supporting the findings of this study are available from the corresponding author upon reasonable request.

## Supplementary Materials

The following supporting information can be downloaded at: https://www.mdpi.com/article/10.3390/jcm15093407/s1, Figure S1: Five-year trends in the main causes of hospital admission; Table S1: Decadal trends in causes of admissions in patients with SLE.

## Abbreviations

The following abbreviations are used in this manuscript:

ACR	American College of Rheumatology
ANOVA	Analysis of variance
APS	Antiphospholipid syndrome
CAPS	Catastrophic antiphospholipid syndrome
CI	Confidence interval
CRP	C reactive protein
DNA	Deoxyribonucleic acid
dsDNA	Double-stranded DNA
EULAR	European Alliance of Associations for Rheumatology
GE	Gerard Espinosa
ICU	Intensive care unit
IQR	Interquartile range
IV	Intravenous
JC-O	Jesús Cívico-Ortega
NS	Non-statistical significance
OR	Odds ratio
SAD	Systemic autoimmune disease
SD	Standard deviation
SDI	Systemic Lupus International Collaborating Clinics Damage Index
SLE	Systemic lupus erythematosus
SLEDAI	SLE Disease Activity Index
SLICC	Systemic Lupus International Collaborating Clinics
