# Supplementary material for: Changing Patterns in Hospitalisations of Patients with Systemic Lupus Erythematosus over Three Decades at a Tertiary Referral Centre in Catalonia
Source: J Clin Med. 2026 Apr 29;15(9):3407. doi: 10.3390/jcm15093407 (PMC13164423; doi:10.3390/jcm15093407)
Supplement: Supplementary file 1 [file jcm-15-03407-s001.zip › Supplementary Table S1.pdf]

Supplementary Table S1. Decadal trends in causes of admissions in patients with SLE.

**Cause of admission**	**1995-2004 n (%)**	**2005-2014 n (%)**	**2015-2024 n (%)**	**p**
SLE Flare	161 (40)	161 (40.1)	104 (25.2)	<0.001
Infection	80 (19.9)	75 (18.7)	129 (31.3)	<0.001
Diagnostic procedures	48 (11.9)	105 (26.2)	114 (27.7)	<0.001
Thrombosis	33 (8.2)	11 (2.7)	6 (1.5)	<0.001
Musculoskeletal	22 (5.5)	9 (2.2)	4 (1)	<0.001
Other SAD	13 (3.2)	4 (1)	17 (4.1)	0.021
Neurological	10 (2.5)	8 (2)	6 (1.5)	0.575
Cardiovascular disease (no thrombotic)	10 (2.5)	10 (2.5)	4 (1)	0.198
Gastrointestinal	6 (1.5)	1 (0.2)	6 (1.5)	0.149
Drug adverse events	4 (1)	2 (0.5)	6 (1.5)	0.385
Hematologic	4 (1)	5 (1.2)	3 (0.7)	0.756
Other	2 (0.5)	5 (1.2)	5 (1.2)	0.475
Oncology	1 (0.2)	4 (1)	4 (1)	0.370
Psychiatry	5 (1.2)	0 (0)	2 (0.5)	0.064
Pregnancy-related	4 (1)	1 (0.2)	0 (0)	0.071
Assessment of disease activity	0 (0)	0 (0)	1 (0.2)	0.377
No specific diagnosis	0 (0)	0 (0)	1 (0.2)	0.377

Abbreviations: SAD: systemic autoimmune disease; SLE: systemic lupus erythematosus.
