# Supplementary figures and images for: Changing Patterns in Hospitalisations of Patients with Systemic Lupus Erythematosus over Three Decades at a Tertiary Referral Centre in Catalonia
Source: J Clin Med. 2026 Apr 29;15(9):3407. doi: 10.3390/jcm15093407 (PMC13164423; doi:10.3390/jcm15093407)

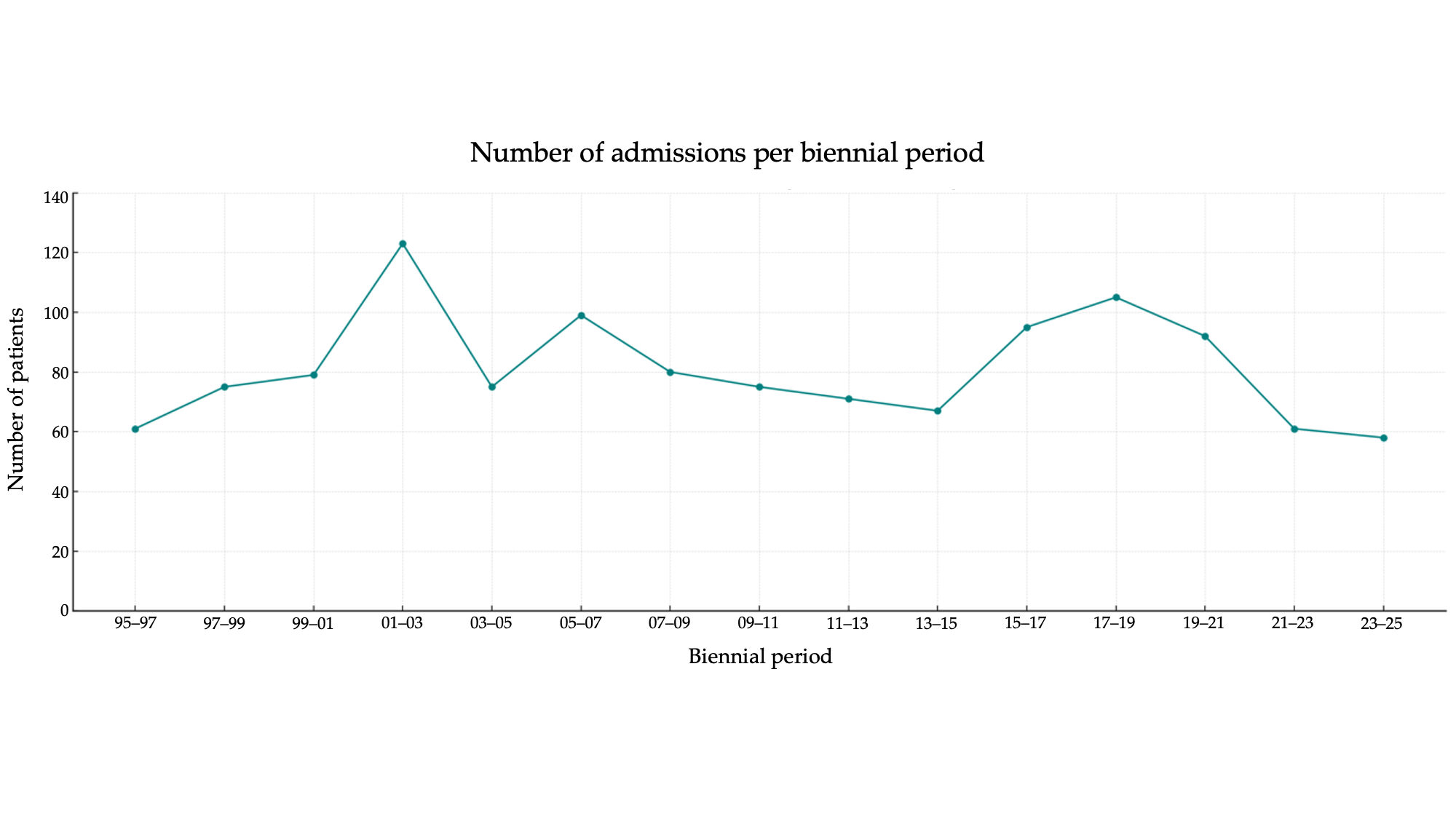

Supplement: Supplementary file 1 [file jcm-15-03407-s001.zip › Supplementary Figure S1.png]
